# Exosomes participate in the alteration of muscle homeostasis during lipid-induced insulin resistance in mice

**DOI:** 10.1007/s00125-014-3337-2

**Published:** 2014-07-30

**Authors:** Hala Aswad, Alexis Forterre, Oscar P. B. Wiklander, Guillaume Vial, Emmanuelle Danty-Berger, Audrey Jalabert, Antonin Lamazière, Emmanuelle Meugnier, Sandra Pesenti, Catherine Ott, Karim Chikh, Samir El-Andaloussi, Hubert Vidal, Etienne Lefai, Jennifer Rieusset, Sophie Rome

**Affiliations:** 1 CarMeN Laboratory (Inserm 1060, INRA 1397, INSA), University of Lyon, Faculté de Médecine Lyon-Sud, Oullins, France; 2 Department of Laboratory Medicine, Clinical Research Center, Karolinska Institutet, Karolinska University Hospital Huddinge, Huddinge, Sweden; 3 Laboratory of Mass Spectrometry, Inserm ERL 1157, CNRS UMR 7203 LBM, Sorbonne Universités, UPMC University Paris 06, CHU Saint-Antoine, Paris, France; 4 Laboratoire Commun de Recherche HCL-bioMérieux Lyon-Sud, Oullins, France; 5 Department of Physiology, Anatomy and Genetics, University of Oxford, Oxford, UK; 6 CarMeN Laboratory (Inserm 1060, INRA 1397, INSA), University of Lyon, Faculté de Médecine Lyon-Sud, Oullins, France

**Keywords:** Exosomes, High-fat diet, Insulin resistance, Muscle, Palmitate

## Abstract

**Aims/hypothesis:**

Exosomes released from cells can transfer both functional proteins and RNAs between cells. In this study we tested the hypothesis that muscle cells might transmit specific signals during lipid-induced insulin resistance through the exosomal route.

**Methods:**

Exosomes were collected from quadriceps muscles of C57Bl/6 mice fed for 16 weeks with either a standard chow diet (SD) or an SD enriched with 20% palm oil (HP) and from C2C12 cells exposed to 0.5 mmol/l palmitate (EXO-Post Palm), oleate (EXO-Post Oleate) or BSA (EXO-Post BSA).

**Results:**

HP-fed mice were obese and insulin resistant and had altered insulin-induced Akt phosphorylation in skeletal muscle (SkM). They also had reduced expression of *Myod1* and *Myog* and increased levels of *Ccnd1* mRNA, indicating that palm oil had a deep impact on SkM homeostasis in addition to insulin resistance. HP-fed mouse SkM secreted more exosomes than SD-fed mouse SkM. This was reproduced in-vitro using C2C12 cells pre-treated with palmitate, the most abundant saturated fatty acid of palm oil. Exosomes from HP-fed mice, EXO-Post Palm and EXO-Post Oleate induced myoblast proliferation and modified the expressions of genes involved in the cell cycle and muscle differentiation but did not alter insulin-induced Akt phosphorylation. Lipidomic analyses showed that exosomes from palmitate-treated cells were enriched in palmitate, indicating that exosomes likely transfer the deleterious effect of palm oil between muscle cells by transferring lipids. Muscle exosomes were incorporated into various tissues in vivo, including the pancreas and liver, suggesting that SkM could transfer specific signals through the exosomal route to key metabolic tissues.

**Conclusions/interpretation:**

Exosomes act as ‘paracrine-like’ signals and modify muscle homeostasis during high-fat diets.

**Electronic supplementary material:**

The online version of this article (doi:10.1007/s00125-014-3337-2) contains peer-reviewed but unedited supplementary material, which is available to authorised users.

## Introduction

Skeletal muscle (SkM)-secreted proteins have important roles in intercellular communications. Among them, a large number of soluble peptide hormones and cytokines (myokines) are capable of triggering adaptations of homeostasis in other peripheral organs or are involved in the process of myogenesis [1]. Studies during the last 10 years have demonstrated that cells also release bioactive nanovesicles. A particularly important class of extracellular vesicle is the exosomes, which represent a discrete population of 50–120 nanometre-sized vesicles [2]. Exosomes are formed in endocytic compartments called multivesicular bodies (MVBs) during endosome maturation, by inward budding of their limiting membrane. They are released from the cell into the microenvironment following the fusion of MVBs with the plasma membrane. Exosomes can transfer both functional protein and RNA species from one cell to another whereby an array of biological processes, including cell proliferation and differentiation, can be affected [3]. It is believed that exosomes could have a much more potent influence on the physiology of the cells they encounter than single-molecule mediators because the numerous proteic, lipidic and nucleic acid components they carry can affect multiple signalling pathways inside target cells. Exosomes can modulate immune-regulatory processes, set up tumour escape mechanisms and mediate regenerative or degenerative processes [4].

All insulin-sensitive tissues release exosomes [5–8]. In this context, it has been demonstrated that adipose tissue-released exosomes in leptin-deficient (*ob*/*ob*) mice are taken up by peripheral blood monocytes and can stimulate the differentiation of monocytes into activated macrophages with increased secretion of TNF-α and IL-6 [9]. This finding suggests that exosomes released from adipose tissue of obese animals could act as a mode of communication with macrophages. Moreover, when administered to wild-type mice, adipose tissue-released exosomes from *ob*/*ob* mice contributed to the development of insulin resistance (IR) [9]. Other studies have shown that adipocyte-derived exosomes are involved in the control of lipid storage and cell size between adipocytes [10] and they have been described as mediators of angiogenesis [11].

Although communication between SkM and other tissues appears to be important in the relationship between obesity and diabetes [12], the possibility that SkM-derived exosomes act as a mode of systemic communication has hitherto never been discussed. Until now, the roles of exosomes in the development of metabolic syndrome and IR are poorly understood and little is known about the connection between lipid exposure and exosome secretion from muscle cells.

Therefore, we investigated whether SkM cells might transmit specific signals via exosomes during lipid-induced IR. Especially, we examined whether exosomes from palmitate-treated myotubes could transfer the deleterious action of palmitate between muscle cells. In addition, we analysed in mice the biodistribution of labelled exosomes from control- or palmitate-exposed myotubes. We provide evidence that exosomes from muscle cells act both as ‘paracrine-like’ signals by locally perturbing muscle homeostasis during lipid-induced IR and as ‘endocrine-like’ signals by targeting other insulin-sensitive tissues in vivo.

## Methods

### Cell culture

C2C12 myoblasts [13] were maintained in DMEM (4.5 g/l glucose, 10% heat-inactivated FBS, 1,000 U/ml penicillin, 1,000 U/ml streptomycin, 2 mmol/l l-glutamine; PAA laboratories; Vélizy-Villacoublay, France) at 37°C under 5% CO_2_ atmosphere. Cell differentiation was induced by replacing FBS with 2% horse serum.

### Mouse model

Male C57BL/6 mice (Harlan Laboratories, Gannat, France) were housed in a common animal centre (PBES - Lyon Sud, Lyon, France). Animal experiments were approved by the Lyon Local Board for Laboratory Animals (No. 2010-008). At 4 weeks, mice were divided into two groups and fed either a standard chow diet (SD; 57% carbohydrate, 5% fat and 18% protein) or an SD diet enriched with 20% palm oil (HP; palmitic acid 44.5%, oleic acid 38.5%). After 16 weeks, blood was withdrawn into heparinised microcapillary tubes from the orbital sinus of anaesthetised mice in a fed state. The mice were then killed by cervical dislocation. Quadriceps and gastrocnemius muscles were excised and used, respectively, for exosome purification or gene and protein expression analyses.

### Palmitate and oleate preparation and C2C12 treatment

**Fig. 1:**
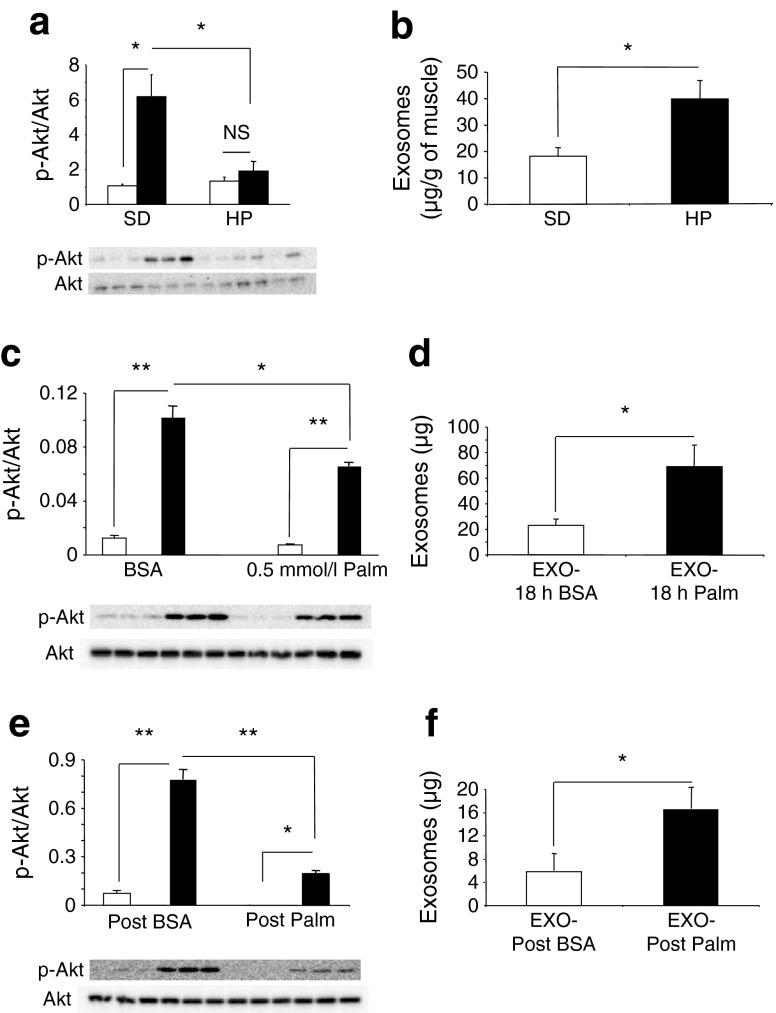
(**a**, **c**, **e**) Phospho-Akt and Akt quantified by western blot in gastrocnemius muscle of SD- and HP-fed mice incubated ex vivo with 10^−7^ mol/l insulin for 15 min (**a**), in C2C12 cells treated with 0.5 mmol/l palmitate (Palm) for 18 h (**c**) and in C2C12 cells 9 h post palmitate treatment (**e**). Data are unitless (phospho-Akt/total Akt intensities). *p < 0.05; **p < 0.01, for indicated comparisons. (**b**, **d**, **f**) Quantities of exosomes released from quadriceps of SD- and HP-fed mice (**b**), from C2C12 cells treated with 0.5 mmol/l palmitate for 18 h (**d**) or from C2C12 cells 9 h post palmitate treatment (**f**). White bars and black bars represent incubation without and with insulin, respectively. *p < 0.05

Palmitate or sodium oleate (P-0500 and Fluka-75165, respectively; Sigma-Aldrich, Saint-Quentin Fallavier, France) were pre-complexed with 10% (wt/vol.) fatty acid-free BSA (Roche Diagnostic, Grenoble, France) in DMEM at 50°C for 2 h to prepare stock solutions of 8 mmol/l. The stock solutions were filtered and diluted in serum-free culture medium to a final concentration of 0.5 mmol/l, as lower concentrations of palmitate or oleate did not affect insulin-induced Akt phosphorylation (see electronic supplementary material [ESM] Fig. 1). The concentration of glucose in the medium (4.5 g/l vs 1 g/l) did not modify alteration of insulin-induced Akt phosphorylation induced by palmitate. For the analysis of insulin signalling, treated myotubes were incubated with or without 10^−7^ mol/l of insulin for 20 min.

### Isolation of muscle exosomes

Exosomes were purified from conditioned media (CM) either from C2C12 cells [14, 15] or from quadriceps cut into small pieces in serum-free DMEM (24 h). Cell debris and organelles were eliminated by centrifugation at 2,000 *g* for 20 min. The supernatant fraction was further centrifuged at 10,000 *g* for 30 min and then filtered through a 0.22 μm filter. Exosomes were pelleted at 100,000 *g* for 70 min at 4°C (Optima L-80-XP ultracentrifuge, type 50-2Ti rotor; Beckman-Coulter, Villepinte, France). Pellets from a single sample were pooled, resuspended in 25 ml PBS and again centrifuged at 100,000 *g* for 70 min. Finally, the exosome pellet was resuspended in 100 μl PBS. Exosomal proteins were quantified using a Bradford protein assay. A NanoSight (Malvern Instruments, Orsay, France) was employed to measure the size of particles [16]. The number of particles and their movement were recorded for 1 × 60 s and analysed using the NS500 software. Exosomes were visualised by transmission electron microscopy as previously described [15] (ESM Fig. 2).

### Production of C2C12 exosomes containing green fluorescent protein

Non-replicative adenoviruses expressing the green fluorescent protein (adeno-GFP) were generated as previously described [17]. Differentiated C2C12 cells were infected with adeno-GFP for 24 h in differentiation medium. Twenty-four hours later all myotubes had green fluorescence in the cytoplasm indicating that all cells had been infected. Myotubes were washed with PBS to remove both non-integrated adenovirus and exosomes from FBS and then were incubated for another 48 h in exosome-depleted DMEM. GFP-containing exosomes accumulated in CM for 48 h were extracted.

Differentiated C2C12 myotubes were incubated with 2 μg GFP-containing exosomes depleted of viral particles (EXO-GFP) per ml of medium [14, 15]. Forty-eight hours later cells were visualised with a Zeiss Axiovert 200 M Fluorescence/Live cell Imaging microscope equipped with Axiovision software (Carl Zeiss, Marly le Roi, France).

### Microarray data analysis

C2C12 RNA profiling was performed using the Mouse GE 4*44K v2 Microarray kit (Agilent Technologies, Les Ulis, France). Briefly, 100 ng of RNA was labelled with Cy-3 using the Low Input Quick Amp Labeling kit (Agilent Technologies) and microarrays were hybridised and scanned following the manufacturer’s instructions. Microarray analyses were performed with R environment using Agi4x44PreProcess and Limma packages from BioConductor [18]. The dataset is available from the GEO database GSE52048 (www.ncbi.nlm.nih.gov/gds/).

### Quantitative real-time PCR

Quantitative real-time PCR (qRT-PCR) was performed using ABsolute QPCR SYBR Green ROX Mix (Abgene, Courtaboeuf, France) with a Rotor-Gene 6000 system (Corbett Life Science, Qiagen, France). Results were normalised with a reference gene encoding TBP (TATA box binding protein) [19]. PCR primers are listed in ESM Table 1.

### Western blotting

Cells were lysed in RIPA lysis buffer (PBS, 0.1% SDS, 0.5% sodium deoxycholate, 1% Nonidet NP40, 5 mmol/l EDTA, 1 mmol/l Na_3_VO_4_, 20 mmol/l NaF, 1 mmol/l dithiothreitol, protease inhibitors). Proteins were denatured and migrated on 12% SDS-PAGE gels and transferred onto nitrocellulose PVDF (polyvinylidene difluoride) membranes. Membranes were incubated with anti-rabbit antibodies (ESM Table 1). Signals were revealed with Immuno detection kit ECL Luminata Classico (Merck-Millipore, Guyancourt, France) and imager Molecular Image ChemiDocXRS+ (Bio-Rad, Marnes-la-Coquette, France). Protein quantification was achieved using ImageLab 3.0 (Bio-Rad).

### Biodistribution of C2C12 exosomes

Exosomes were pelleted in the presence of 1 μmol/l fluorescent lipophilic tracer DiR (1,1-dioctadecyl-3,3,3,3-tetramethylindotricarbocyanine iodide) (D12731; Invitrogen Life Technologie, Cergy Pontoise, France) by ultracentrifugation (110,000 *g*, 90 min). DiR dye has been used previously for both cell and exosomal tracking in vivo [20]. A washing step was performed by re-suspending the pellet in 25 ml PBS and ultracentrifuging (110,000 *g*, 90 min). The pellet was resuspended in 210 μl PBS. Particle analysis was performed with a NanoSight (Malvern Instruments [16]) to measure the particle size distribution of post re-pelleting with DiR. DiR-labelled exosomes were injected through the tail vein of female C57BL/6 mice (16 g/body weight) for 24 h to obtain the highest amount of fluorescence in each tissue. As a control we injected with mock-PBS-DiR, and performed the same procedures as for the labelling of exosomes but without exosomes (ESM Fig. 3). Mice were then killed and the organs were carefully harvested and imaged for 2 s (exCitation 710 nm, emission 760 nm) using a high-sensitivity CCD camera IVIS Spectrum (PerklinElmer, Sverige, Sweden). As shown in ESM Fig. 3, the free DiR showed a signal, with a total tissue signal about 14–15% that of C2C12-DiR-EXO.

Animal experiments were approved by the Swedish Local Board for Laboratory Animals and performed in accordance with ethical permission and designed to minimise animal suffering and pain.

### Exosome lipidomic analysis

The fatty acid profile of total exosome lipids was determined by fatty acid methyl ester (FAME) analysis. Total lipid extract was trans-esterified in 2 ml of methanol H_2_SO_4_ 2% at 70°C for 1 h. FAMEs were analysed by GC-MS on an Agilent 5975 in series with GC (Hewlett Packard 6890 series) (Agilent Technologies). Quantification was achieved by normalisation with an internal standard of nonadecanoic acid methyl ester and the response factors for the various fatty acids were calculated with weighted methyl ester calibrators FAME mix (Restek, Lisses, France) [21].

### Impedance measurement with the xCELLigence RTCA DP Instrument

The effect of exosomes on C2C12 myoblast proliferative capacities was monitored with the xCELLigence live cell analysis System (Life Science Roche, Meylan, France), which offers dynamic live cell monitoring [22]. One day post-plating, cells were grown in serum-free DMEM and 4.5 g/l glucose supplemented with exosomes, and monitored every 15 min for 24 h [15]. At the end of the experiment, cells were trypsinised, counted and their sizes were determined by using the Scepter 2.0 handheld automated cell counter from Millipore (Guyancourt, France).

### Statistical analysis

All results were expressed as mean ± SEM. A parametric Student’s *t* test was used for mean comparison. A *p* value < 0.05 was considered significant.

## Results

### Lipids modified muscle exosome release

Mice fed with HP for 16 weeks were obese, hyperinsulinaemic and hyperglycaemic (ESM Fig. 4) compared with SD-fed mice. Insulin-induced Akt phosphorylation was altered ex vivo in gastrocnemius muscles of HP-fed mice (Fig. 1a). Muscles of HP- and SD-fed mice were incubated ex vivo for 24 h in serum-free medium and exosomes released into the CM during this time period were extracted by filtration and ultracentrifugation [5, 14, 23] (ESM Fig. 2). The vesicle size distribution displayed a bell-shaped curve (ESM Fig. 2) in agreement with the reported size of exosomes [2, 24]. As shown on Fig. 1b, muscles from HP-fed mice secreted more exosomes than the muscles from SD-fed mice. This result was reproduced in-vitro when C2C12 cells were incubated for 18 h in serum-free medium with palmitic acid only (C16:0), the most abundant saturated fatty acid of palm oil (Fig. 1c, d). This tendency was still observed 9 h post-palmitate incubation compared with cells pre-treated with BSA (Fig. 1e, f). Reducing the concentration of palmitate in the C2C12 medium from 0.5 mmol/l to 0.1 mmol/l led to a decrease in exosomes released (80 μg of exosomes are released from C2C12 treated with 0.5 mmol/l palmitate compared with 30 μg when cells are treated with 0.1 mmol/l palmitate).

### Exosomes can transfer proteins between C2C12 myotubes

**Fig. 2:**
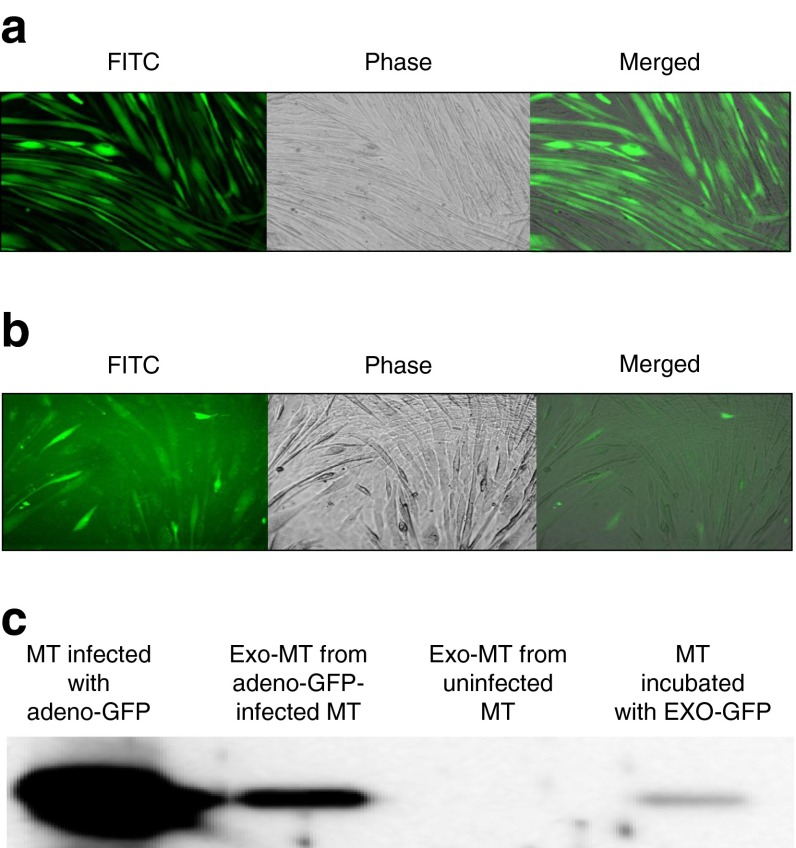
(**a**) C2C12 myotubes (MT) infected with adeno-GFP (magnification × 10). (**b**) MT incubated for 48 h with EXO-GFP released from infected MTs described in (**a**) (magnification × 20). (**c**) Western blot detection of GFP in infected MTs (1 μg) or in their released EXO-GFP (1 μg) or in MTs incubated with EXO-GFP (50 μg); Exo-MT, exosomes from myotubes

As we recently demonstrated that exosomes could transfer microRNAs (miRNAs) and proteins between muscle cells [14, 15] we aimed to determine whether myotubes could use the exosomal route to transfer proteins. Myotubes were infected with adeno-GFP protein (Fig. 2a) in order to collect EXO-GFP. Fresh C2C12 myotubes were incubated with EXO-GFP and fluorescence in their cytoplasm was monitored. Forty-eight hours post-incubation, myotubes with fluorescent cytoplasm were detected (Fig. 2b) indicating that exosomes had transferred their GFP contents into recipient myotubes (Fig. 2c). This data suggested that increased release of exosomes from muscle cells incubated with palmitic acid might modify the crosstalk between muscle cells and thus perturb muscle homeostasis

### EXO-Post Palm increased the Akt content of recipient muscle cells

**Fig. 3:**
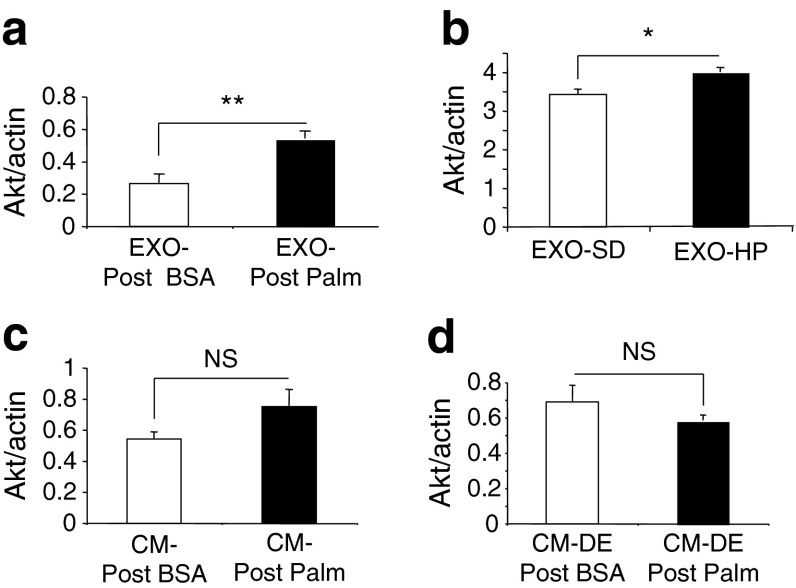
Akt quantified by western blot. Actin was used as loading control. Data are unitless (total Akt/actin intensities). (**a**, **b**) C2C12 myotubes incubated with C2C12-released exosomes collected from cells pre-treated with EXO-Post Palm vs EXO-Post BSA (**a**) or with quadriceps-released exosomes from HP-fed mice vs SD-fed mice (**b**). (**c**, **d**) C2C12 myotubes incubated with CM of cells pre-treated with palmitate (CM-Post Palm) (**c**) or with CM-Post Palm depleted of exosomes (CM-DE) (**d**)

To determine whether palmitate treatment affected the biological properties of muscle-released exosomes, differentiated myotubes were incubated with exosomes from cells pre-treated with BSA or palmitate [14, 15]. To ensure that exosomes were not contaminated with cell-free palmitate we used exosomes collected 9 h after palmitate removal from the medium (EXO-Post Palm). As shown in Fig. 1e, insulin-induced Akt phosphorylation was still altered during this time period, indicating that the effect of palmitate was not lost. Results indicated that incubation with EXO-Post Palm, compared with EXO-Post BSA, induced an increase in the total Akt content of the recipient myotubes (Fig. 3a). An increase in the Akt level was also found when examining quadriceps-released exosomes from HP-fed mice (EXO-HP) vs SD-fed mice (EXO-SD) (Fig. 3b). By contrast, EXO-Post Palm and EXO-HP did not significantly affect insulin-stimulated Akt phosphorylation (data not shown), indicating that they did not transfer lipid-induced insulin-resistance between muscle cells.

To determine the specific effects of exosomes compared with the global medium from palmitate-treated C2C12 cells, conditioned medium (CM-Post Palm) was divided during exosome extraction. Although not significant, CM-Post Palm induced an increase in the total Akt level (Fig. 3c) as did the exosomal fraction alone. CM-Post Palm depleted of exosomes had no effect on Akt level (Fig. 3d), indicating that exosomes likely participated in the effect of CM on total Akt in C2C12 cells.

### EXO-Post Palm modified expression of genes involved in cell cycle and muscle differentiation in recipient myotubes

**Fig. 4:**
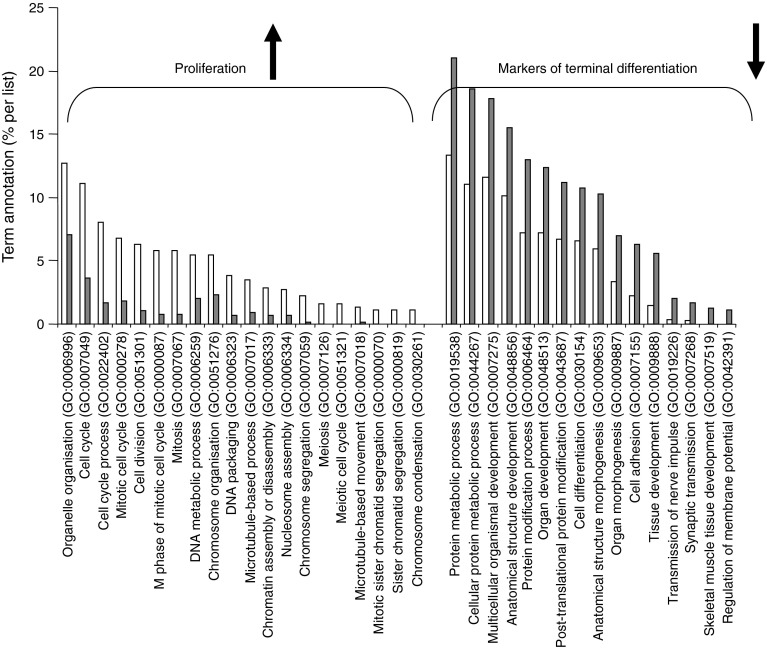
Gene ontology (GO) pathways (levels from 3 to 9) significantly enriched in genes differentially regulated in C2C12 cells treated with EXO-Post Palm vs EXO-Post BSA (*p* < 0.05). White bars, upregulated genes; grey bars, downregulated genes

**Fig. 5:**
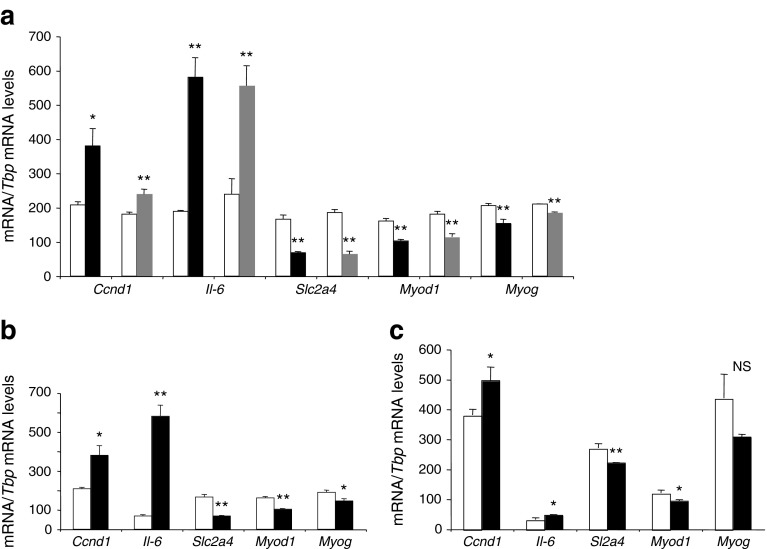
Expression levels of *Ccnd1*, *Il*-*6*, *Slc2a4*, *Myod1* and *Myog* quantified by qRT-PCR. (**a**) C2C12 cells pre-treated with EXO-Post BSA (white bars), EXO-Post Palmitate (black bars) or EXO-Post Oleate (grey bars). (**b**) C2C12 cells pre-treated with palmitate (black bars) or BSA (white bars). (**c**) mRNA expressions in gastrocnemius muscle of SD (white bars) or HP (black bars) mice. All data are unitless (mRNA/*Tbp* mRNA level). *n* = 4 biological replicates; *p < 0.05, **p < 0.01 vs EXO-Post BSA, BSA or SD

Myotubes were incubated either with EXO-Post BSA or EXO-Post Palm in serum-free medium for 24 h and their transcriptomes were analysed by microarrays. mRNA levels of 240 genes were significantly modified in response to EXO-Post Palm (163 genes were upregulated and 77 were downregulated) compared with EXO-Post BSA (ESM Table 2). Functional enrichment analyses (Babelomics 4.3), comparing the 163 upregulated genes with the 77 downregulated genes, revealed that ‘cell cycle’, ‘mitosis’ and ‘DNA metabolic process’ genes were significantly enriched in the upregulated genes (Fig. 4). The 77 significantly downregulated genes were involved in ‘cell adhesion’, ‘cell differentiation’, ‘development’ and ‘protein metabolic process’ (Fig. 4). Microarray data were confirmed by measuring *Ccnd1*, *Il*-*6* and *Slc2a4* (*Glut4*) mRNA levels (ESM Table 2 and Fig. 5a). In addition, we found a downregulation of *Myog* and *Myod1*, two markers of muscle cell terminal differentiation, in cells treated with EXO-Post Palm (Fig. 5a).

### EXO-Post Palm affected C2C12 proliferation

**Fig. 6:**
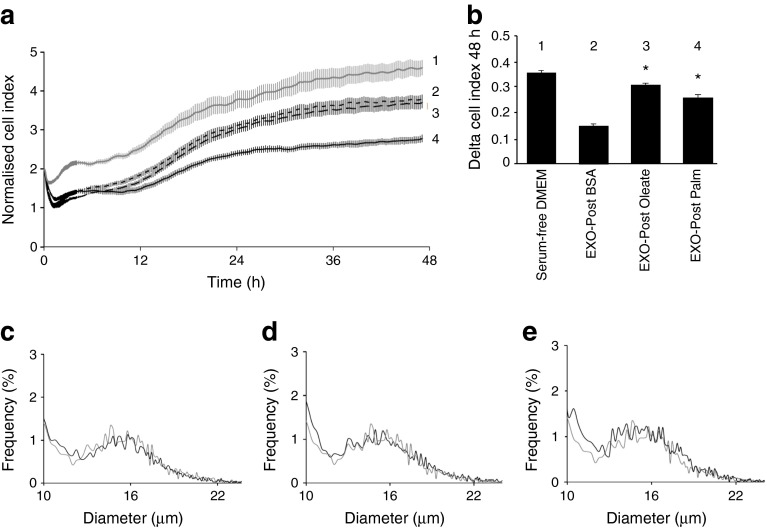
C2C12 myoblasts (*n* = 8) were incubated with serum-free DMEM supplemented with EXO-Post Palm or EXO-Post Oleate. (**a**, **b**) Impedance measurement with the xCELLigence System. (**a**) The presence of the cells on top of the electrodes will affect the local ionic environment at the electrode/solution interface, leading to an increase in the electrode impedance. The more cells attached to the electrodes, the larger the increase in electrode impedance. (**b**) Data is graphed as ‘Delta Cell Index’, which is the change in impedance from the time point immediately after cells were added (*p < 0.05 vs EXO-Post BSA). (**c**–**e**) Myoblast sizes determined by the Scepter 2.0 handheld automated cell counter after 48 h proliferation. (**c**) Serum-free DMEM (grey line) or EXO-Post BSA (black line). (**d**) EXO-Post BSA (grey line) or EXO-Post Palm (black line). (**e**) EXO-Post BSA (grey line) or EXO-Post Oleate (grey line)

As the biological effect of exosomes was studied on unproliferative C2C12 myotubes, we wondered whether the increase in *Ccnd1* mRNA level involved in the regulation of cell cycle resulted from a residual myoblast population. Myoblasts were grown in serum-free medium complemented with EXO-Post BSA or EXO-Post Palm. The cell growth curves were automatically recorded every 15 min for 48 h. As shown in Fig. 6a, b, EXO-Post Palm induced myoblast proliferation compared with EXO-Post BSA. These effects were not associated with modification of mean cell sizes (Fig. 6c–e).

### Exosomes transferred lipids between muscle cells

**Fig. 7:**
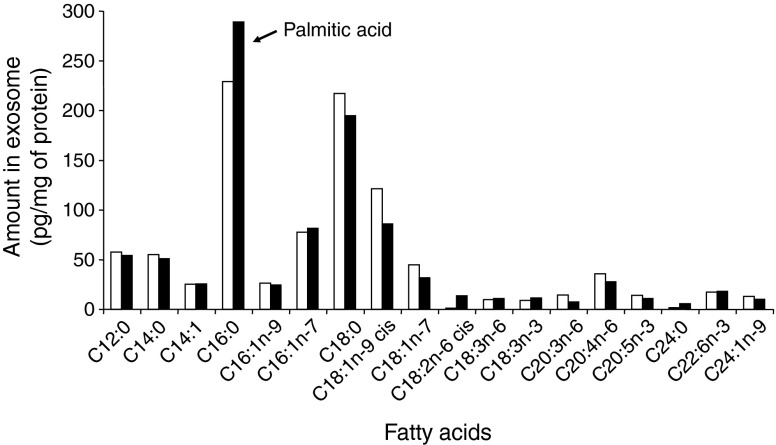
Quantification of fatty acids in exosomes. EXO-Palm (black bars) and EXO-BSA (white bars) from C2C12 treated for 18 h with BSA or palmitate

We observed that *Slc2a4*, *Myod1*, *Myog*, *Ccnd1* and *Il*-*6* mRNA levels were altered in the same direction both in muscle cells used to collect exosomes (muscle cells of HP-fed mice vs SD-fed mice; C2C12 cells treated with palmitate vs BSA) (Fig. 5b, c) and in the recipient C2C12 cells treated with those exosomes (Fig. 5a). From these data we suspected that exosomes from palmitate-treated cells could potentially transfer the effect of palmitate by transferring lipid messengers between cells. We performed a lipidomic analysis to determine whether treatment with palmitate had modified the exosome composition. Exosomes released from cells treated with palmitate were enriched in palmitate compared with exosomes from cells treated with BSA (Fig. 7), suggesting that EXO-Post Palm could transfer palmitate between muscle cells. To determine whether such transfer might occur with other fatty acids, we incubated C2C12 myotubes with exosomes collected from cells pre-treated with oleate (EXO-Post Oleate), the most abundant unsaturated fatty acid of palm oil. In contrast with palmitate, oleate did not alter insulin-stimulated Akt phosphorylation in muscle cells (ESM Fig. 1). However, compared with EXO-Post BSA, EXO-Post Oleate altered the expression of *Ccnd1*, *Il*-*6*, *Slc2a4*, *Myod1* and *Myog* in C2C12 myotubes in a similar manner to EXO-Post Palm (Fig. 5a). EXO-Post Oleate also induced myoblast proliferation (Fig. 6).

### Biodistribution of exosomes from muscle cells

**Fig. 8:**
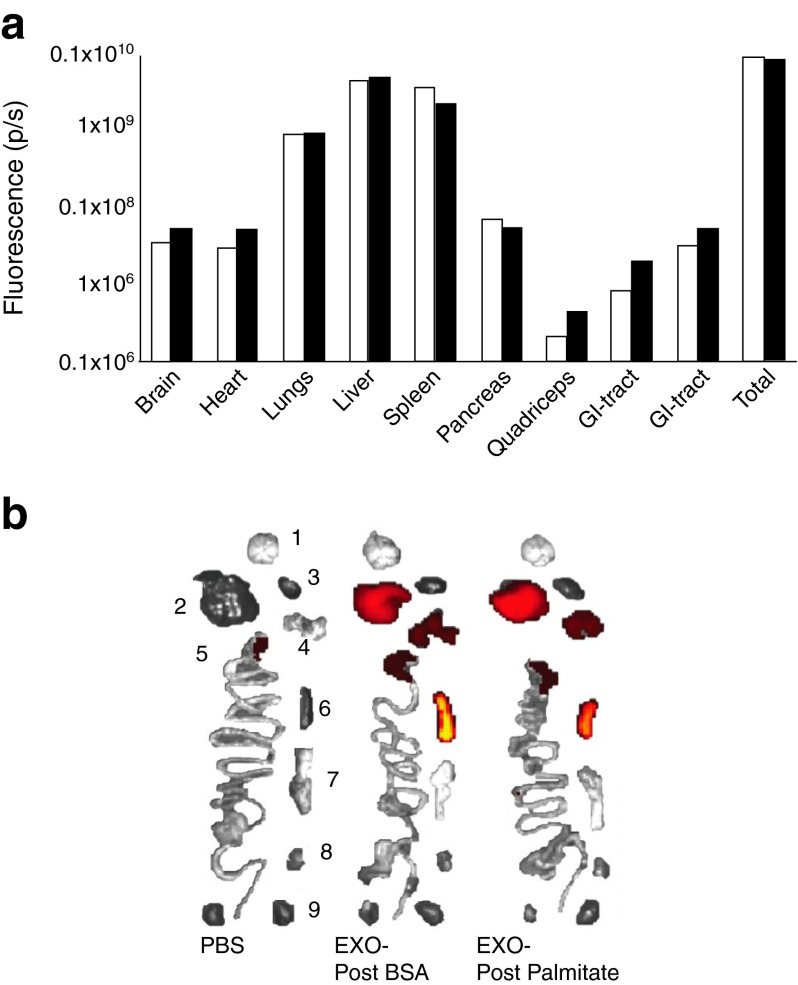
Biodistribution of C2C12 exosomes. EXO-Post BSA (6.02 × 10^10^ particles/ml; white bars) and EXO-Post palmitate (3.44 × 10^10^ particles/ml; black bars) were used for tail injection in C57Bl/6 mice. (**a**) Quantification of fluorescent signals for each organ, normalised to background. (**b**) Organs targeted by C2C12 DiR exosomes; 1 = brain, 2 = liver, 3 = heart, 4 = lungs, 5 = gastrointestinal tract, 6 = spleen, 7 = pancreas, 8 = quadriceps, 9 = kidney

EXO-Post Palmitate or EXO-Post BSA labelled with fluorescent lipophilic dye DiR were injected into the tail vein of mice. Twenty-four hours post-injection, the mice were killed and fluorescence was monitored in various tissues. Figure 8 shows that most of the exosomes went to the liver, spleen and lungs but fluorescence was detected in all analysed tissues. Both types of exosome (BSA vs palmitate) targeted the same tissue.

## Discussion

Palmitic acid is the most abundant systemic saturated NEFA of palm oil and has received considerable attention in investigations of metabolic consequences of dietary lipids since it is suspected to participate in the onset of IR in SkM [25, 26]. In this context it has been demonstrated that palmitate-induced IR in C2C12 myotubes is associated with impaired expression of myokines (i.e. irisin, CTRP15 [family with sequence similarity 132, member B] and FGF21 [fibroblast growth factor 21]) known for their potential beneficial roles in metabolic diseases [27]. In agreement, irisin in plasma is reduced in type 2 diabetes by 50% [28]. These data indicated that under chronic elevation of plasma NEFA SkM may secrete specific signals that would act either as ‘paracrine-like’ signals by locally perturbing muscle homeostasis or as ‘endocrine-like’ signals by targeting other insulin-sensitive tissues. In this study, HP-fed mice consuming a standard diet enriched with 20% palm oil for 16 weeks presented marked obesity, hyperinsulinaemia and hyperglycaemia compared with SD-fed mice. Moreover, they displayed altered Akt phosphorylation in response to insulin and reduced expression of *Slc2a4* mRNA in SkM. Interestingly, HP-fed mice were also found to have reduced mRNA levels of *Myod1* and *Myog*, two markers of muscle differentiation, and increased mRNA level of *Ccnd1*, indicating that palm oil had a deep impact on muscle homeostasis in addition to IR.

The first finding of our study is that feeding mice with palm oil modified the rate of exosome secretion from SkM ex vivo. This was confirmed in vitro by showing that increasing the amount of palmitate in the medium of C2C12 cells induced an increase in exosome release. It is possible that de novo synthesis of ceramides from palmitate could contribute to an increase in intracellular exosome formation as exosome membranes are enriched in ceramides and intracellular ceramide levels are involved in exosome secretion by permitting the inward budding of MVBs [29, 30]. Here, we did not quantify ceramides in exosomes but we found a marked enrichment in palmitic acid after treatment of C2C12 cells indicating that there was a modification of the lipid composition of exosomes. In addition, palmitate-induced exosome release could also occur through the palmitoylation of proteins involved in invagination of endosomal membranes (i.e. proteins involved in the ESCRT [endosomal sorting complex required for transport] machinery [31]), possibly leading to modifications of the proteins’ subcellular localisation, stability, trafficking, translocation to lipid rafts, aggregation and interactions with new effectors [32].

Although palmitate treatment does not reproduce exactly the in vivo situation where a mixture of fatty acids exists provided by the diet, these results support the notion that excessive concentration of circulating saturated fatty acids might modify exosome secretion from SkM in vivo.

We have previously shown that muscle-released exosomes are involved in the process of myogenesis, being able to transfer proteins and miRNAs from differentiated myotubes to proliferating myoblasts [14, 15]. In this study we further demonstrated that exosomes can also transfer their protein contents between differentiated muscle cells. Thus we postulated that in response to palm oil, muscle cells might secrete exosomes able to transfer part of the deleterious effect of palm oil to neighbouring cells. Our data showed that palmitate-induced IR is not transferred between muscle cells through the exosomal route. However, EXO-Post Palm, as well as EXO-HP, was able to perturb the myotube phenotype (i.e. decrease in *Myog* and *Myod1* mRNA levels) and to induce myoblast proliferation (i.e. increase in Akt protein and *Ccnd1* mRNA levels). Interestingly, these alterations in genes expressions were similar to those observed in vivo in muscles of HP-fed mice or in palmitate-treated C2C12 cells. These results indicate, therefore, that muscle-released exosomes can likely transfer the deleterious effect of palm oil between muscle cells by transferring lipids and thus may participate in vivo in the alterations of muscle homeostasis in high-fat diets.

EXO-Post Palm or EXO-HP modified the expression of genes encoding 20 secreted proteins in recipient myotubes (ESM Table 2). Among them, *Cxcl1* and *Cxcl5* have been found to be altered in the serum of patients with type 2 diabetes [33, 34]. We validated the upregulation of *Il*-*6*, the gene encoding IL-6, one of the several pro-inflammatory cytokines associated with IR and type 2 diabetes [35]. In vitro studies have suggested that IL-6 plays a part in myogenesis and muscle atrophy [36, 37]. Thus these data support the concept that in addition to altering muscle homeostasis, muscle-exosomes are able to modulate the expression of several myokines, hence suggesting a potential role for these extracellular vesicles in whole-body homeostasis during lipid-induced IR.

Until now the mechanism of exosome uptake by target cells has been poorly understood. Integrins and tetraspanins [38], as well as cell-surface heparan sulfate proteoglycans, have been implicated in exosome uptake and internalisation [39]. However, despite the fact that exosomes can be used for the delivery of small RNAs and anti-inflammatory agents to target cells in vivo [40, 41] it is not known whether exosomes have restricted tissue specificities. Indeed, in these previous studies exosomes were modified in order to target tissues of interest. We have shown recently that, in vitro, myotube-released exosomes can transfer siRNA and miRNA to myoblasts [14]. Importantly, we found in the present study that fluorescently labelled muscle-released exosomes intravenously injected into mice were incorporated into various tissues in addition to SkM. Incorporation into the pancreas and liver might suggest that SkM could transfer specific signals through the exosomal route to key metabolic tissues in vivo. Alterations in this signalling system during a high-fat diet may eventually contribute to the development of IR and type 2 diabetes. Further studies are now required to demonstrate whether exosomes from insulin-resistant SkM can reach the blood circulation and regulate the homeostasis of the other insulin-sensitive tissues and to decipher the affected functions in these target tissues.

## Electronic supplementary material

Below is the link to the electronic supplementary material.

ESM Fig. 1 (PDF 228 kb)

ESM Fig. 2 (PDF 15051 kb)

ESM Fig. 3 (PDF 49 kb)

ESM Fig. 4 (PDF 76 kb)

ESM Table 1 (PDF 14 kb)

ESM Table 2 (XLS 85 kb)

## Abbreviations

adeno-GFP: Adenoviruses expressing GFP
CM: Conditioned medium
FAME: Fatty acid methyl ester
GFP: Green fluorescent protein
HP: SD enriched with 20% palm oil
IR: Insulin resistance
miRNA: MicroRNA
MVB: Multivesicular body
qRT-PCR: Quantitative real-time PCR
SD: Standard chow diet
SkM: Skeletal muscle
