# Supplementary figures and images for: Exosomes participate in the alteration of muscle homeostasis during lipid-induced insulin resistance in mice
Source: Diabetologia. 2014 Jul 30;57(10):2155–64. doi: 10.1007/s00125-014-3337-2 (PMC4153976; doi:10.1007/s00125-014-3337-2)

## ESM Figure 1

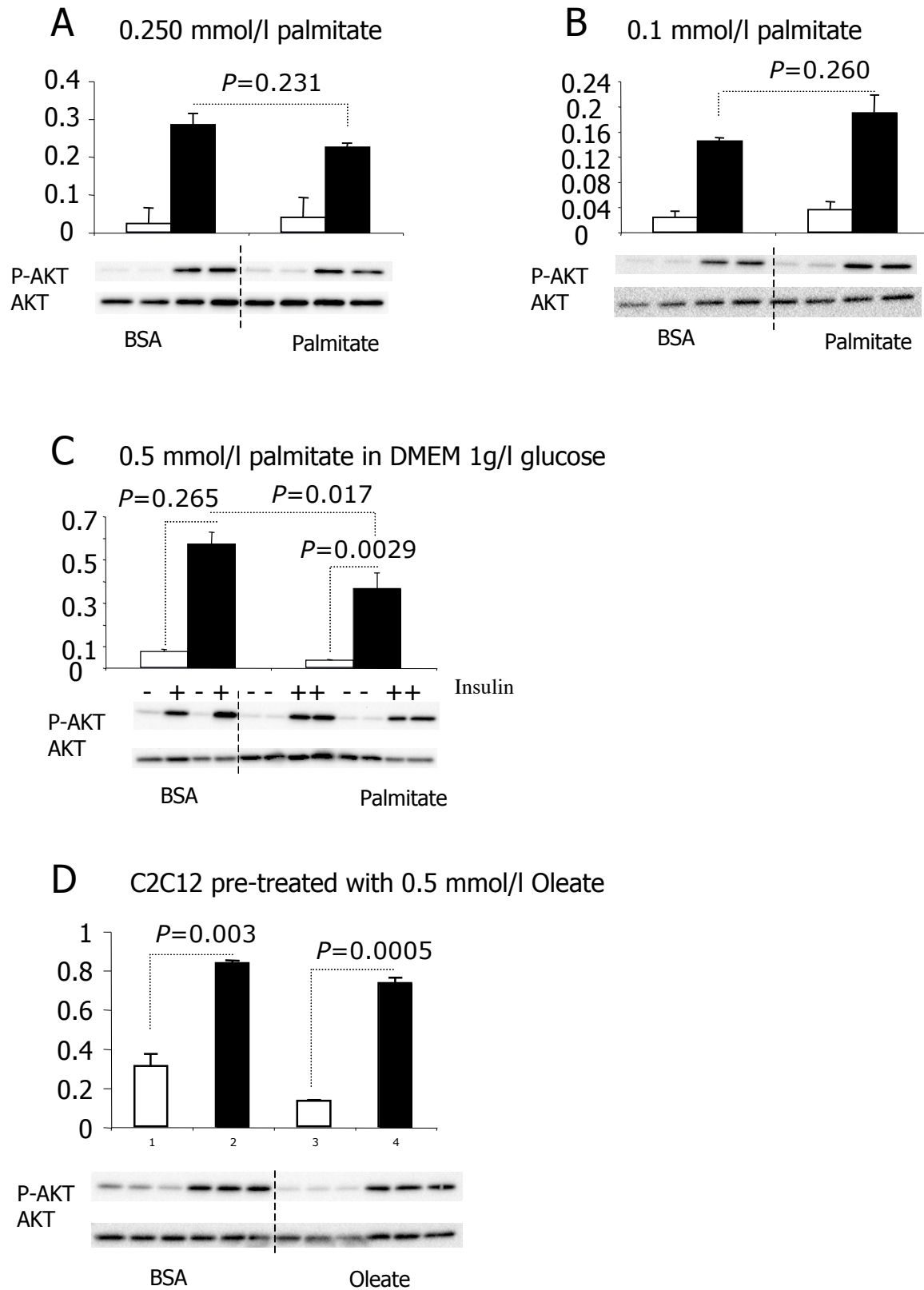

ESM Figure 1 : Phospho-AKT and AKT quantified by WB in C2C12 cell extracts.

Supplement: Supplementary file 1 — (PDF 228 kb) [file 125_2014_3337_MOESM1_ESM.pdf]
