# Supplementary material for: Exosomes participate in the alteration of muscle homeostasis during lipid-induced insulin resistance in mice
Source: Diabetologia. 2014 Jul 30;57(10):2155–64. doi: 10.1007/s00125-014-3337-2 (PMC4153976; doi:10.1007/s00125-014-3337-2)

ESM Figure 2

A_ Transmission electron microscopy images. Bars=100nm (left) or 50nm (right).

C2C12 exosomes (Exo-MT) muscle exosomes (Exo-Quad)

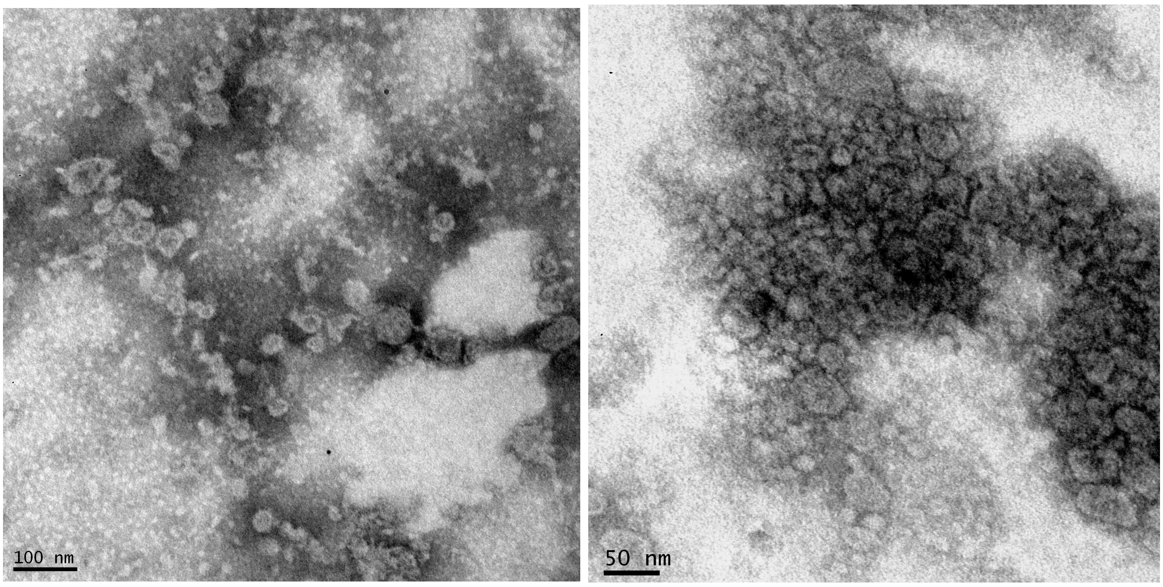

B_Western-blot analyses of representative exosomal proteins

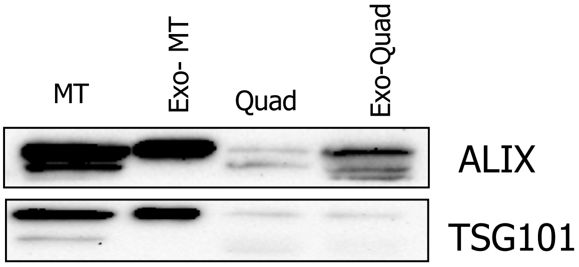

MT=C2C12 myotubes (50µg)
Exo-MT=exosomes from C2C12 myotubes (2.5µg)
Quad=*Quadriceps* (50µg)
Exo-Quad=exosomes from *Quadriceps* (25µg)

C_Size distribution of muscle-released vesicles.

C2C12 exosomes (Exo-MT) muscle exosomes (Exo-Quad)

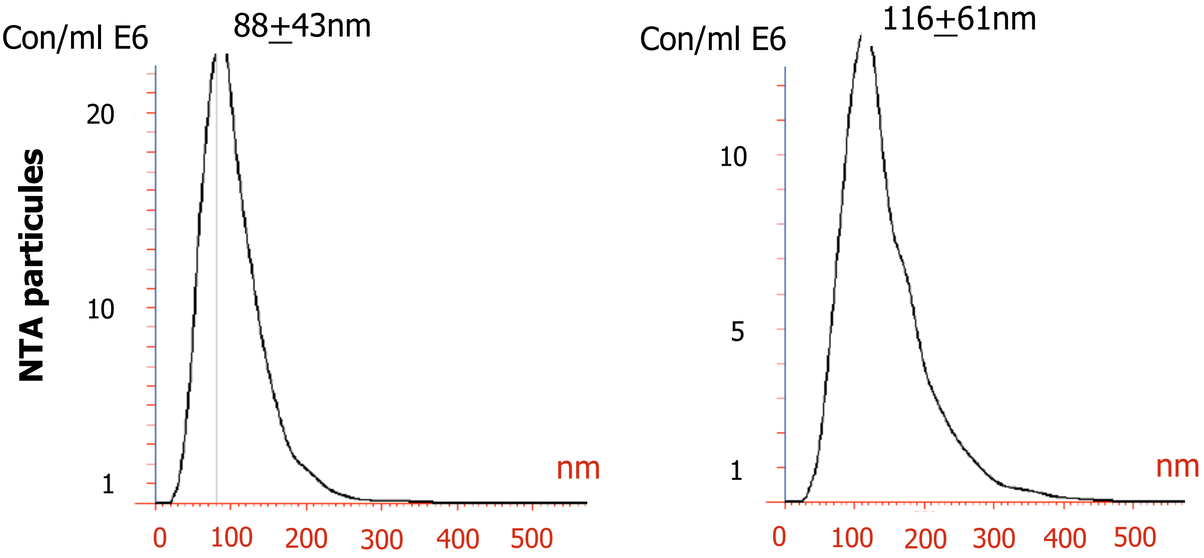

Supplement: Supplementary file 2 — (PDF 15051 kb) [file 125_2014_3337_MOESM2_ESM.pdf]
