# Supplementary material for: Exosomes participate in the alteration of muscle homeostasis during lipid-induced insulin resistance in mice
Source: Diabetologia. 2014 Jul 30;57(10):2155–64. doi: 10.1007/s00125-014-3337-2 (PMC4153976; doi:10.1007/s00125-014-3337-2)
Supplement: Supplementary file 3 — (PDF 49 kb) [file 125_2014_3337_MOESM3_ESM.pdf]

ESM Figure 3

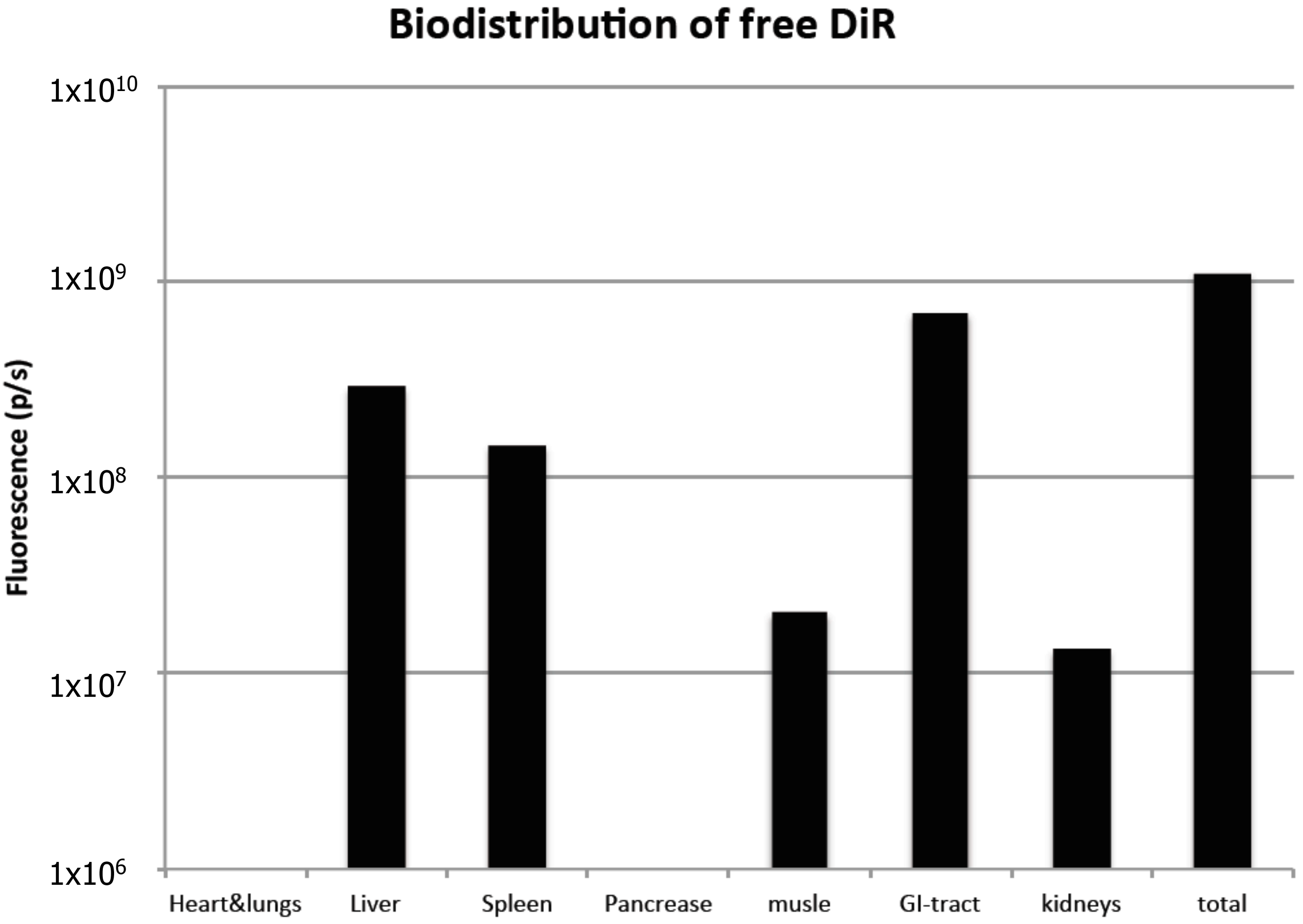

The mock-PBS-DiR was done as the labeling of exosomes but without exosomes.
25 ml PBS with 1 uM DiR was ultracentrifuged at 110, 000g for 90 min
(no extra washing step, to have as much free DiR as possible as control).
The pellet was resuspended in 200 ul PBS and injected in a mouse.
As shown, the distribution pattern is very different than with exosomes,
with majority of the signal originating from the GI-tract.
