# Supplementary material for: Exosomes participate in the alteration of muscle homeostasis during lipid-induced insulin resistance in mice
Source: Diabetologia. 2014 Jul 30;57(10):2155–64. doi: 10.1007/s00125-014-3337-2 (PMC4153976; doi:10.1007/s00125-014-3337-2)
Supplement: Supplementary file 4 — (PDF 76 kb) [file 125_2014_3337_MOESM4_ESM.pdf]

ESM Figure 4

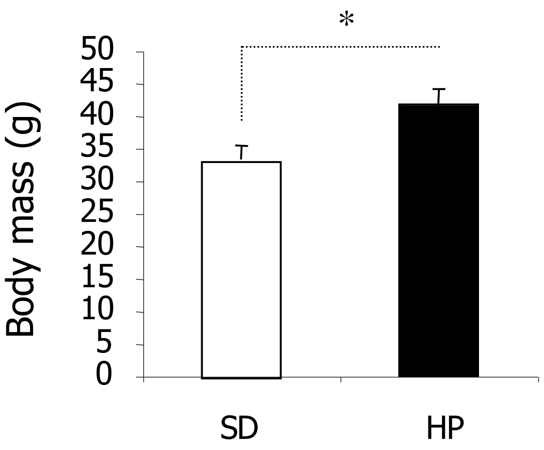

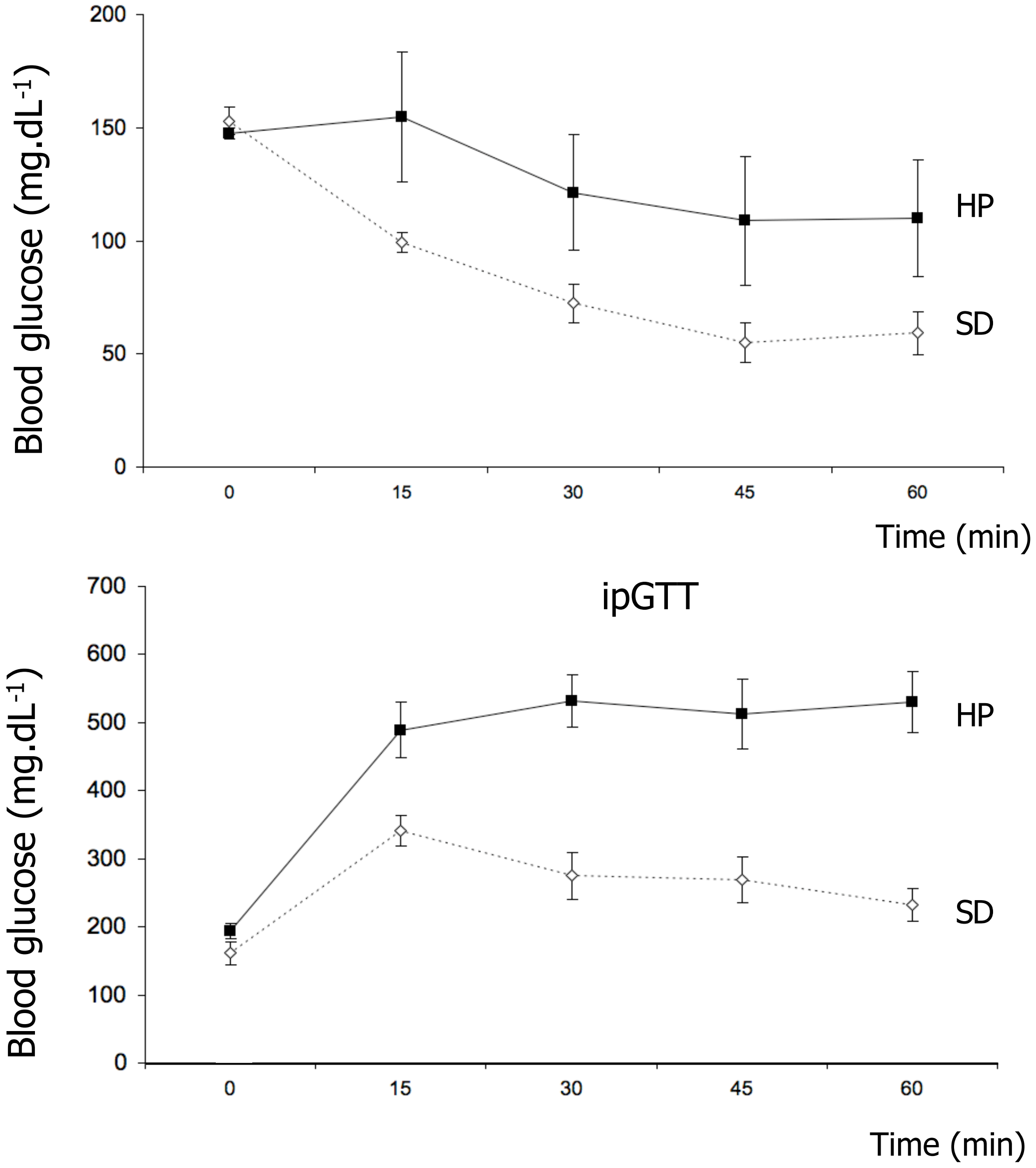

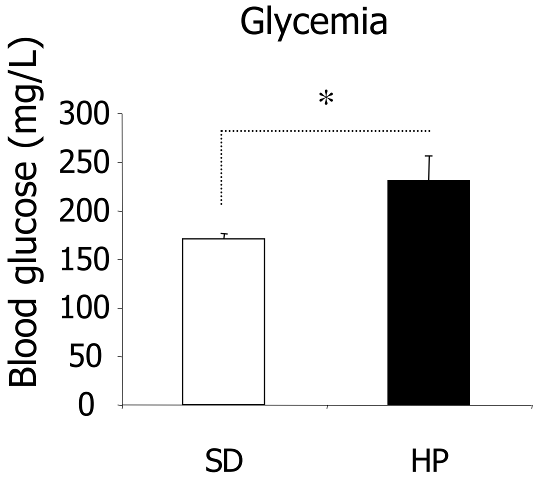

Mice metabolic parameters after 16 weeks SD or HP diets. (A,B) Body mass and glycemia measured before sacrifices unde fed state. (C) i.p.ITT after 15 weeks of diet in a 6-hours fasted state. Blood glucose concentrations measured before and 15, 30, 45 and 60 min after insulin *i.p.* injection (0.75U/kg) (n=5). (D) i.p.GTT performed after 16 weeks of diet in a 6-hours fasted state. Blood glucose concentrations measured before and 15, 30, 45 and 60min after glucose *i.p*. injection (2g/kg) (n=5).
