# Supplementary material for: Exosomes participate in the alteration of muscle homeostasis during lipid-induced insulin resistance in mice
Source: Diabetologia. 2014 Jul 30;57(10):2155–64. doi: 10.1007/s00125-014-3337-2 (PMC4153976; doi:10.1007/s00125-014-3337-2)
Supplement: Supplementary file 5 — (PDF 14 kb) [file 125_2014_3337_MOESM5_ESM.pdf]

Supplementary Table 1: A_PCR primers and B_Western-blot antibodies used in this study

A

Gene Names	Gene Symbols	Sens Primers	Antisens Primers
TATAbox binding protein	*Tbp*	TTCACATCACAGCTCCCCAC	TGGTGTGCACAGGAGCCAAG
Cyclin D1	*Ccnd1*	CTTCCTCTCCAAAATGCCAG	TGGAGGGTGGGTTGGAAATG
Myogenin	*Myog*	CAACCCAGGAGATCATTTGC	CATATCCTCCACCGTGATGC
Interleukin-6	*Il-6*	AGTTGCCTTCTTGGGACTGAT	TCCACGATTTCCCAGAGAAC
Myogenic differentiation 1	*Myod1*	TCCAGCCCGCGCTCCAACTGC	TCGACACGGCCGCACTCTTCC
Solute carrier family 2 (facilitated glucose transporter), member 4	*Glut4*	GGGTTTCCAGTATGTTGCGG	CTGGGTTTCACCTCCTGCTC

B

Antibodies	Antibody references	Company names
Alix	sc-49268	Santa Cruz
TSG101	sc-6037	Santa Cruz
CD81	sc-166028	Santa Cruz
beta-Actin	A5060	Sigma Aldrich
Phospho Akt ser-473	4060	Cell signaling
total Akt	9272	Cell signaling
Horseradish Peroxidase conjugated secondary antibody	172-1019	Bio-Rad
